# Super-Resolution Ultrasound Imaging of Renal Vascular Alterations in Zucker Diabetic Fatty Rats during the Development of Diabetic Kidney Disease

**DOI:** 10.3390/diagnostics13203197

**Published:** 2023-10-12

**Authors:** Stinne Byrholdt Søgaard, Sofie Bech Andersen, Iman Taghavi, Mikkel Schou, Christina Christoffersen, Jens Christian Brings Jacobsen, Hans Martin Kjer, Carsten Gundlach, Amy McDermott, Jørgen Arendt Jensen, Michael Bachmann Nielsen, Charlotte Mehlin Sørensen

**Affiliations:** 1 Department of Biomedical Sciences, University of Copenhagen, 2200 Copenhagen, Denmark; stinnebyrholdt@gmail.com (S.B.S.); posttilsofiebech@gmail.com (S.B.A.); christina.christoffersen@regionh.dk (C.C.); jcbrings@sund.ku.dk (J.C.B.J.); amy.mcdermott@sund.ku.dk (A.M.); 2 Department of Diagnostic Radiology, Rigshospitalet, 2100 Copenhagen, Denmark; mbn@dadlnet.dk; 3 Center for Fast Ultrasound Imaging, Department of Health Technology, Technical University of Denmark, 2800 Lyngby, Denmark; imat@dtu.dk (I.T.); jaje@dtu.dk (J.A.J.); 4 BK Medical, 2730 Herlev, Denmark; mikkel.schou@ge.com; 5 Department of Clinical Biochemistry, Rigshospitalet, 2100 Copenhagen, Denmark; 6 Department of Applied Mathematics and Computer Science, Technical University of Denmark, 2800 Lyngby, Denmark; hmkj@dtu.dk; 7 Department of Physics, Technical University of Denmark, 2800 Lyngby, Denmark; cagu@fysik.dtu.dk; 8 Department of Clinical Medicine, University of Copenhagen, 2200 Copenhagen, Denmark

**Keywords:** ultrasound localization microscopy, diagnostic imaging, type 2 diabetes, chronic kidney disease, renal injury

## Abstract

Individuals with diabetes at risk of developing diabetic kidney disease (DKD) are challenging to identify using currently available clinical methods. Prognostic accuracy and initiation of treatment could be improved by a quantification of the renal microvascular rarefaction and the increased vascular tortuosity during the development of DKD. Super-resolution ultrasound (SRUS) imaging is an in vivo technique capable of visualizing blood vessels at sizes below 75 µm. This preclinical study aimed to investigate the alterations in renal blood vessels’ density and tortuosity in a type 2 diabetes rat model, Zucker diabetic fatty (ZDF) rats, as a prediction of DKD. Lean age-matched Zucker rats were used as controls. A total of 36 rats were studied, subdivided into ages of 12, 22, and 40 weeks. Measured albuminuria indicated the early stage of DKD, and the SRUS was compared with the ex vivo micro-computed tomography (µCT) of the same kidneys. Assessed using the SRUS imaging, a significantly decreased cortical vascular density was detected in the ZDF rats from 22 weeks of age compared to the healthy controls, concomitant with a significantly increased albuminuria. Already by week 12, a trend towards a decreased cortical vascular density was found prior to the increased albuminuria. The quantified vascular density in µCT corresponded with the in vivo SRUS imaging, presenting a consistently lower vascular density in the ZDF rats. Regarding vessel tortuosity, an overall trend towards an increased tortuosity was present in the ZDF rats. SRUS shows promise for becoming an additional tool for monitoring and prognosing DKD. In the future, large-scale animal studies and human trials are needed for confirmation.

## 1. Introduction

Diabetes mellitus affected more than 500 million adults globally in 2021 and is associated with micro- and macrovascular diseases [1]. Roughly 95% of these people suffer from type 2 diabetes (T2D) [2]. Diabetic kidney disease (DKD) is one of the leading causes of kidney failure, reported to cause up to ~40% of the cases in 2009 [3,4,5]. The rate of DKD progression varies between the types of diabetes; T2D has a more heterogenic development due to the influence of body mass index, genetics, sex, and ethnicity, making the identification of persons at risk challenging [6]. In humans, two clinical parameters are used to assess the progression of DKD: estimated glomerular filtration rate (eGFR) and albuminuria. Furthermore, histopathological features can confirm a DKD diagnosis, but a biopsy is only performed when the disease’s course is unusual [7]. Despite an absence of albuminuria and low eGFR, histopathological changes can still occur, suggesting that DKD may develop imperceptibly [8,9]. Microvascular changes such as rarefaction may precede a decline in eGFR [10]. Additionally, increased microvascular tortuosity may develop following nephron loss in correlation with renal scarring, i.e., tubulointerstitial fibrosis progression [11,12]. As renal microvascular alterations may precede the current clinically detectable onset of DKD, an additional monitoring method is needed to visualize and quantify the renal microvasculature.

In 2015, super-resolution ultrasound (SRUS) imaging was developed, enabling the visualization of the microvasculature in vivo through the vascular injection of ultrasound contrast made of microbubbles (MBs) [13]. SRUS significantly improved image resolution by breaking the diffraction limit of conventional ultrasound, facilitated by the subsequent post-processing procedure of the acquired ultrasound data. The MBs are imaged and then isolated, detected, localized, and tracked to create MB trajectories as surrogates for vessels [14,15]. By imaging the MBs for a period of several minutes, it is possible to create a 2D image of the renal vascular bed, visualizing vessels below 75 µm.

SRUS has been used in humans for the visualization of brain pathology, for instance, but also tumor characterization [16,17,18]. Recently, SRUS showed a superiority to ultrasound in the evaluation of human kidney allograft vascularization [19]. Additionally, SRUS has been used on rodent kidneys to detect decreased vascular density after acute kidney injury and flow-velocity changes [20,21,22,23]. However, altered renal vascular morphology as a consequence of DKD development has not previously been investigated using SRUS.

The Zucker diabetic fatty (ZDF) rats are widely used for studies on T2D, as they develop diabetes at 8–12 weeks of age [24,25,26]. Early renal damage can be detected as an increased albumin-to-creatinine ratio at week ~30, and, by weeks 35–37, irreversible DKD can be detected histologically as defined by glomerulosclerosis [27,28,29].

This study aimed to investigate the possibility of detecting a decreased renal vascular density and an increased renal vessel tortuosity in vivo prior to an increase in albuminuria in the ZDF rats using SRUS. The SRUS imaging was substantiated through comparisons with ex vivo micro-computed tomography (µCT) and histopathological examinations.

## 2. Materials and Methods

### 2.1. Ethical Considerations

The experiments were performed at the Department of Biomedical Sciences at the University of Copenhagen, and the procedures were executed according to license 2020-15-0201-00547, approved by the Danish Animal Experiments Inspectorate under the Ministry of Food, Agriculture and Fisheries of Denmark. Forty-four rats (Charles River, Écully, France) were obtained at 5–9 weeks of age, fed a Purina 5008 diet (LabDiet, St. Louis, MO, USA) ad libitum, and allowed free access to water. They were housed in the local animal facility at the University of Copenhagen, in the Department of Experimental Medicine. Due to ethical concerns regarding their health, eight of the obtained rats (five ZDF rats and three control rats) were euthanized prior to inclusion in the experiments. Consequently, the study was performed on 36 rats in total, with a slight overweight of control rats. Per the ARRIVE guidelines, the sample size was determined to prevent an overuse of animals, as a power calculation was not possible to perform due to unknown expected variance in outcome [30].

### 2.2. Study Overview

**Figure 1:**
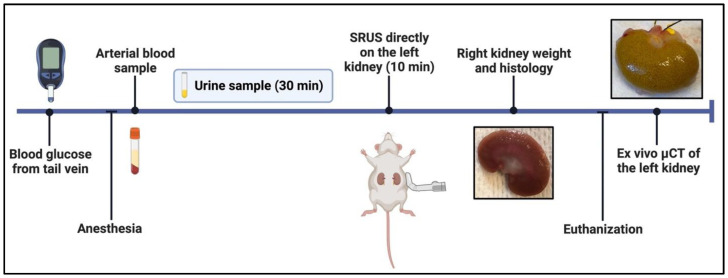
Overview of the experimental sequence. Before the rat was anesthetized, blood glucose from the tail vein was measured. After anesthesia, a blood sample via a catheter in the carotid artery as well as urine collection via a catheter in the ureter were acquired. The 10 min SRUS imaging was performed on the left kidney. Just before the animal was euthanized, the right kidney was excised for histology. Finally, the left kidney was perfused with CT contrast and excised to be scanned. Figure created with BioRender.com, accessed February 2023.

The experiments were conducted on six 12-week-old, five 22-week-old, and six 40–43-week-old male ZDF rats compared with nineteen age-matched male lean Zucker rats (LZRs). The specific time points for the experiments were chosen to visualize the development of structural changes over time, based on the previously reported literature: 12 weeks of age, at the onset of T2D characterized by hyperglycemia; 22 weeks of age, during the initiation of DKD, prior to a significant increase in the albumin-to-creatinine ratio (ACR); and 40 weeks of age, when irreversible glomerulopathy is present in the histopathological examination, defining late stage DKD [24,27,28,29,31]. Figure 1 shows an overview of the experimental sequence, and each step is described in the following text.

### 2.3. Animal Preparation

Blood glucose was measured on the blood from the tail vein of the conscious animals using the Accu-Chek Aviva system (Roche Diabetes Care, Inc., Indianapolis, IN, USA). The rats were then anesthetized in an induction chamber with 5% of isoflurane delivered in 65% of nitrogen and 35% of oxygen, and a 2% of isoflurane concentration upholding the anesthesia was delivered through a tracheostomy. Throughout the experiment, the ventilation rate was 69 respirations/min, controlled using a mechanical ventilator (Ugo Basile, Gemonio, Italy), and the mean arterial pressure (MAP) was measured with a Statham P23-dB pressure transducer (Gould, Oxnard, CA, USA) via a polyethylene catheter (PE-50) in the left carotid artery. To prevent voluntary respiration, Nimbex (0.85 mg/mL, GlaxoSmithKline, Brentford, UK) was continuously infused at 20 µL/min via a polyethylene catheter (PE-10) in the left jugular vein, controlled with a syringe pump (SP210iw, WPI, Sarasota, FL, USA). A laparotomy was performed, and a metal retractor was placed below the left diaphragm to expose the left kidney and reduce movements, primarily caused by the ventilation. An arterial blood sample was collected, added to 10 µL of EGTA, and centrifuged at 7000 rpm/g for 10 min. Urine was then collected over 30 min using a catheter in the left ureter (PE-10 into a PE-50).

### 2.4. In Vivo Super-Resolution Ultrasound Imaging and Post-Processing

The SRUS imaging was performed directly on the left kidney using a modified BK5000 scanner and an X18L5s hockey stick transducer (both from BK Medical, Herlev, Denmark) [32]. The kidney was covered with air-free gel, and the probe was oriented in a longitudinal view of the kidney, presenting both the renal cortex and medulla. To minimize motion, the probe was fixed with a holder (Quipu srl, Pisa, Italy) during the 10 min acquisition time. The ultrasound contrast agent Sonovue (Bracco, Milan, Italy) was intravenously infused using a 1:20 dilution at a rate of ~55 µL/min (syringe pump: SP210iw, WPI, Sarasota, FL, USA) via a catheter (PE-10) in the jugular vein. Furthermore, a custom-built device turned the syringe by 180 degrees every 10 s to ensure a continuous influx of MBs. Contrast-enhanced images were obtained with a frame rate of 55 Hz, a center frequency of 10 MHz, and a mechanical index of 0.1. The modification of the scanner enabled long acquisitions (10 min) of amplitude modulation sequences interleaved with B-mode sequences (for motion compensation), as well as the live streaming of the data to a disk for post-processing. The amplitude modulation sequence enhanced the non-linear signal of the MBs and enabled their extraction from the more linear signals of the tissue. Before MB tracking, the MB positions were motion-corrected using speckle tracking to estimate the displacement on a reference image [32]. The MB trajectories were created with a hierarchical Kalman tracker by linking the MB positions that belonged to the same MB [33]. Only the MBs observed in a minimum of three consecutive frames or more were considered a track. The final SRUS image was created by accumulating all the MB trajectories in one single 2D image.

After the SRUS scan, the right kidney was excised, weighed, decapsulated, and prepared for histology by fixation in 4% formaldehyde for 24 h.

### 2.5. Ex Vivo µCT Imaging

The rats were subsequently prepared for the infusion of the intravascular ex vivo µCT contrast agent Microfil (MV122, Flow Tech Inc., Carver, MA, USA). To isolate the renal circulation and improve contrast flow into the left kidney, ligatures were placed around the left renal artery and vein, around the cranial and the caudal part of the abdominal aorta, respectively. The rats were then intravenously heparinized with 1000 IE/kg of Heparin (“SAD” 1000 IE/mL, Amgros, Copenhagen, Denmark). The aortic catheter (PE-50) was retrogradely inserted and fixed with the catheter tip just below the left renal artery, and the cranial aortic ligature was tightened. The ligature around the renal vein closest to the inferior vena cava was closed, and a hole was made in the renal vein to allow the infused solutions to leave the renal vasculature freely. The left kidney was flushed with 9 mL of preheated (40 °C) heparinized saline (1000 IE/mL heparin diluted 1:100 in isotonic saline) at 2 mL/min, controlled with a pump (SP210iw, WPI, Sarasota, FL, USA). Then, the rats were euthanized by decapitation. When only clear saline exited the renal vein, the rats were moved to a fume hood, where ~3 mL of Microfil was infused at 1 mL/min. When the entire surface of the kidney was yellow and the contrast had left the renal vein, the ligatures around the renal vein and artery were closed. To secure contrast curing, the kidney was left for 60 min and then excised, decapsulated, and fixed in 4% of formaldehyde for at least 24 h. Finally, the kidney was embedded in paraffin in a customized holder. The kidneys were collected and scanned the same week after each sub-experiment (12, 22, and 40 weeks). The kidneys were scanned individually for 11 h in a ZEISS XRadia 410 Versa µCT scanner (Carl Zeiss Microscopy GmbH, Jena, Germany) with an isotropic voxel size of 26.5 µm and the following additional settings: tube voltages of 40 kV, a power of 10 W, a 0.175 mA current, an appertaining LE2 filter, a 360° scan around the vertical axis with 3201 different projections (0.112° rotation steps).

### 2.6. Kidney Histology

The right kidney was stained with periodic acid-Schiff (PAS) to evaluate the degree of glomerulopathy [34]. A longitudinal section of one kidney per animal was examined for affected glomeruli using conventional light microscopy with a 4x objective (Leitz, Wetzlar, Germany). The affected glomeruli were defined by the presence of sclerotic areas, microaneurysms, or glomerular atrophy and qualitatively graded as follows: grade 1 (minimal) had none or few glomeruli affected; grade 2 (moderate) had some glomeruli affected; and grade 3 (severe) had numerous glomeruli affected [28,29]. The morphological evaluation was carried out on all the glomeruli throughout the cortex to prevent selection bias and was executed by an experienced anatomist blinded to the samples.

### 2.7. Measurements of Metabolic Parameters

To estimate glomerular filtration rate as an indicator of renal function, urine and plasma creatinine concentrations were measured to calculate creatinine clearance. Cobas reagents (Roche Diagnostics, Basel, Switzerland) were used for the creatinine (CREP2: 03263991). The same calibrator (Cfas:10759350) was used throughout all the assays, and controls for the urine (U1: BioRad 397, U2: BioRad 398) and the plasma (K1: BioRad 697, K3: BioRad 699) were used. All the assays were manually designed for a 96-well format and measured in duplicates. To determine the glomerular damage, urine albumin concentrations were measured (ELISA Kit ab108789, Abcam, Cambridge, UK) and the albumin-to-creatinine ratio (ACR) was calculated.

### 2.8. Quantification of Vascular Structural Alterations in the SRUS Images

**Figure 2:**
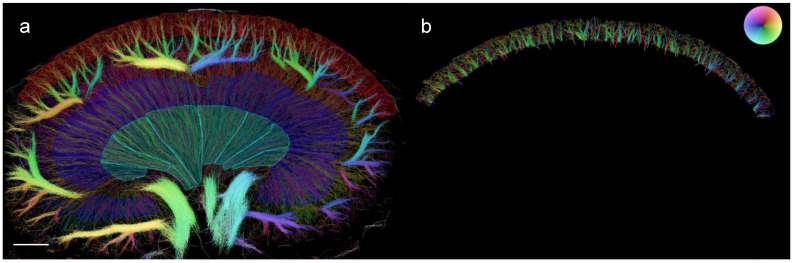
Segmentation of a rat kidney separated into cortex, outer medulla, and inner medulla in a SRUS image. (**a**) The red represents the cortex, the dark blue the outer medulla, and the turquoise area represents the inner medulla. (**b**) The vessel tracks in the cortex after regional separation. The microbubble flow directions are illustrated by the color wheel in the upper right corner, e.g., green trajectories in the cortex representing cortical radial veins. Scale bar on (**a**): 2 mm.

The segmentation of the anatomical kidney regions (cortex (CO), outer medulla (OM), and inner medulla (IM)) was performed manually by placing the regions of interest (ROIs) on the SRUS images using a built-in graphical user interface in MATLAB (MathWorks, Natick, MA, USA) (Figure 2a). This allowed the isolation of specific kidney regions, most importantly the CO (Figure 2b). The outer delineation of the arcuate arteries/veins and the surface of the kidney defined the CO region [35]. The OM region was defined by a dense vasa recta, as these vessels lie in bundles and transition into a less dense vasa recta in the IM region, from where they continue unbranched to the tip of the papilla [36]. Additionally, the outer stripe of the outer medulla was not labeled, as the vasa recta extend to the cortical side of the arcuate arteries/veins; the bottom half of the CO region, closest to the hilum, was not labeled due to presence of a visually less dense vasculature which is likely not representative [37].

The vascular density and tortuosity were measured within the selected ROIs. The vascular density was measured in automatically placed 50% overlapping 2 × 2 mm^2^ patches. Local density was measured in each patch as track-filled pixels versus all pixels. This metric was given a representative value ranging from zero to one, with one representing a patch completely covered with tracks. The mean vascular densities were subsequently converted into percentages. Vessel tortuosity was measured using the distance metric calculated as the actual track path divided by the shortest distance from the start to the end of the track [38]. This metric was given a representative value ranging from one to infinity, with a higher value equating a greater tortuosity.

### 2.9. µCT Processing and Quantification of Vascular Density

A quantitative and qualitative assessment of µCT was performed to substantiate our SRUS findings. The full volume of the µCT scans of six rats (a randomly selected subset: an LZR and a ZDF rat from each age group) were processed using ITK-snap (version 3.8.0) with the following two steps: (1) delineation of the kidney boundary and the cortex-to-medulla boundary, and (2) segmentation of the vessel structures and estimation of the local vessel radii [39]. The inner and outer medulla were combined for the µCT measurement; thus, the segmentation was performed differently than on the SRUS images, as the renal cortex was our primary focus and the segmentation on the µCT images was time-consuming. Three people completed the segmentation, two of them blinded to the type of rats to minimize performance bias. The third person could not be blinded as she was the primary investigator of the study.

The anatomical division of the kidney into the cortex and medulla regions was obtained through the following three steps. Firstly, a mask separating the kidney from the background was generated using a semi-automatic threshold-based region-growing algorithm. Manual editing was carried out when needed, e.g., to create a smooth closing at the hilum. Secondly, a mask delineating the inner part of the cortex at the arcuate vessels was created manually. In practice, approximately every fifth coronal slice was segmented using a polygon tool. The segmentation in the remaining slices was filled using label interpolation. Finally, a third mask representing the cortex region was created by ‘subtracting’ the medulla from the entire kidney mask. In the second step, an intensity threshold was manually set to segment all the vascular structures. The threshold was tuned for each scan to obtain a threshold that neither included the area between vessels (too low) nor excluded too many vessels (too high). Afterward, the local radius of the segmented vessels was estimated using the Porespy Python library [40].

**Figure 3:**
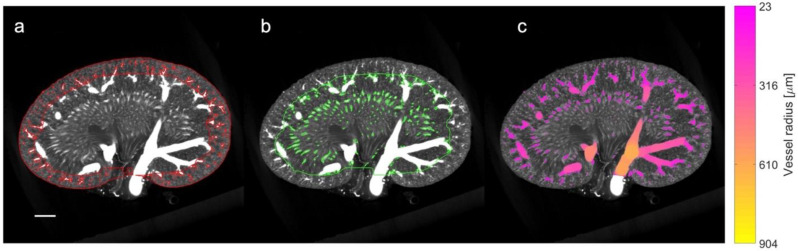
µCT processing and density analysis. The coronal slice of a µCT scan is portrayed from a 22-week-old lean Zucker rat with the following: (**a**) the cortex defined by red borders and the mask of small vessels included in the cortical vascular density; (**b**) the medulla defined by the green border and the mask of small vessels included in the density analysis for the medulla region (note the exclusion of the large segmental and arcuate vessels); and (**c**) colored voxels representing the full vessel segmentation and the subsequent vessel radius estimation. The color indicates the local vessel radius in accordance with the colormap to the right. Size bar: 2 mm.

The density of the vessel structures was estimated separately for the cortical (Figure 3a) and medullary regions (Figure 3b) using MATLAB and calculated by taking the ratio of the vessel volume (within the region) to the full volume of the region. The analysis excludes the influence of the larger vessels, i.e., the renal artery and vein, the segmental vessels, and the arcuate vessels. This was facilitated by thresholding the estimated vessel radius map to divide the vessel segmentation into ‘small’ and ‘large’ vessel masks (Figure 3c). For the cortex region the threshold was set to a vessel radius of 10 voxels (approx. 230 µm). Similarly, for the medulla region, the threshold was set to 6 voxels (approx. 140 µm). The region of large vessels was then removed to calculate the density.

The exclusion of the large vessels was carried out differently in the µCT and in the SRUS. On the SRUS images, the areas with large vessels were not included in the labeling. Therefore, to increase similarity in the density measurements conducted in the SRUS and in the µCT, the large vessels were excluded from the µCT measurements.

### 2.10. Statistical Analyses

Two-way ANOVA was used to test differences in metabolic parameters and the quantified parameters measured from the SRUS images within and between the three age groups. Pairwise multiple comparisons were made using Sidak’s multiple comparisons test. All the statistical analyses and graphs were made using GraphPad Prism (version 9.3.1 for Mac, GraphPad Software, San Diego, CA, USA).

## 3. Results

### 3.1. Metabolic Parameters

**Figure 4:**
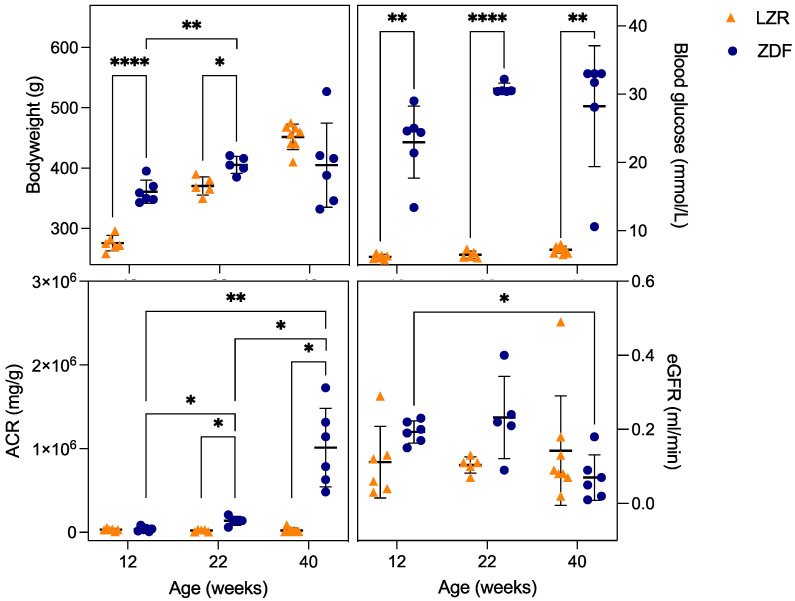
Bodyweight, blood glucose, albumin-to-creatinine ratio (ACR), and creatinine clearance (eGFR). The displayed parameters measured at the study end in the lean Zucker rats (LZRs) and the Zucker diabetic fatty rats (ZDF) at an age of 12, 22, and 40 weeks. The orange triangles represent the lean rats and the blue circles the rats with diabetes. A significant difference is indicated by *p* < 0.05, * 0.01–0.05, ** 0.001–0.01, and **** <0.0001. The lines indicate mean ± SD.

As illustrated in Figure 4 and Table 1, the body weight of the 12- and 22-week-old ZDF rats were significantly increased compared to the age-matched lean controls (*p* < 0.001 and *p* = 0.02, respectively). No significant difference was found in the 40-week-old group as the ZDF rats’ weight decreased or stagnated, similarly to what is found in the literature [27,41]. The blood glucose in the ZDF rats was significantly higher than in the lean rats at each time point, representing hyperglycemia from week 12 (*p* = 0.002). The initiation of DKD was confirmed at week 22 by a significantly increased ACR compared to the measurement in lean rats (*p* = 0.02). No significant differences in the creatinine clearance (GFR) between groups were found; however, a significant decrease in the GFR was found from week 12 to 40 in the ZDF rats (*p* = 0.01). There was a non-significant tendency in the ZDF rats towards an increased GFR in the early stages (12 and 22 weeks) compared to the lean group, possibly representing hyperfiltration in the early stage of DKD, which was replaced by a decline in filtration associated with glomerular atrophy at 40 weeks [42,43]. As detailed in Table 1, the kidney weight was significantly increased in the ZDF rats (*p* < 0.01) compared to the control rats; this was explained by hypertrophy in both the glomerular and tubular cells in the diabetic kidneys [44]. The mean arterial pressure (MAP) was similar between the anesthetized LZR and the ZDF rat in the three different age groups, with a lower MAP in the ZDF rats compared to the LZRs’ at 40 weeks of age, similar to what is found in the literature [41]. The diuresis significantly increased with age in the ZDF rats (*p* < 0.02), correlated with the progression of diabetes, as hyperglycemia leads to elevated filtered glucose, generating osmotic diuresis when excreted [45].

### 3.2. Glomerulopathy Grading

**Figure 5:**
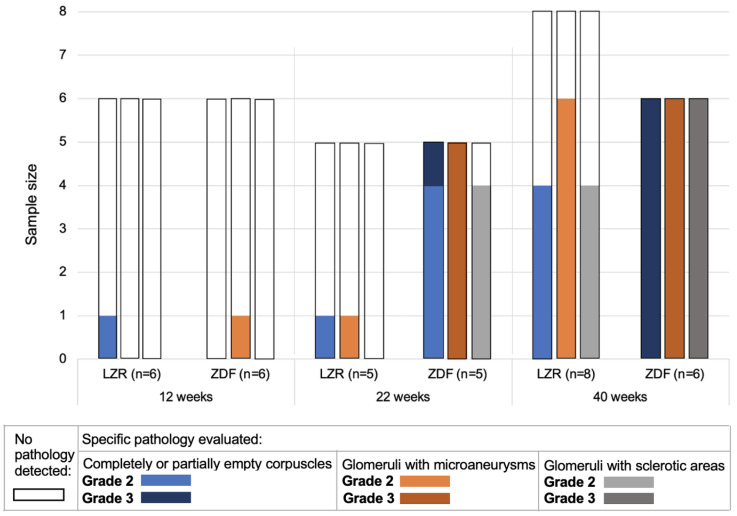
Histological evaluation of glomeruli. Qualitative grading of glomerulopathy in PAS-stained kidney tissue from the lean Zucker rats (LZRs) and the Zucker diabetic fatty rats (ZDF) by week 12, 22, and 40. Grade 2 is displayed in light colors with respect to the specific pathology evaluated and is defined as the specimens with moderate involvement. Grade 3 is displayed in dark colors and is defined as the number of specimens with severe involvement.

**Figure 6:**
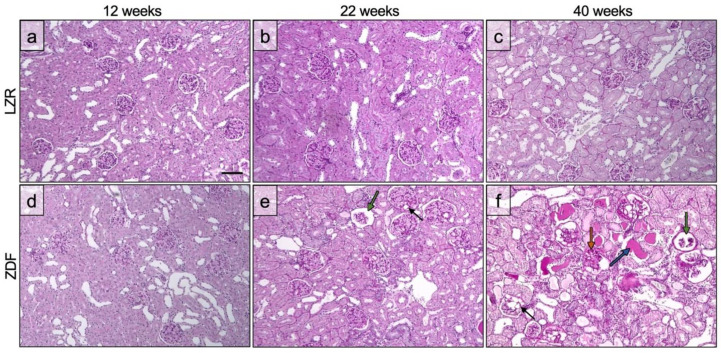
PAS-stained kidney tissue with a section of the cortex. Each age group is represented in the lean Zucker rats (LZRs) and the Zucker diabetic fatty rats (ZDF). (**a**–**d**) shows the glomeruli without any pathology. (**e**) displays microaneurysms (black arrow) and glomerular atrophy (green arrow). (**f**) presents microaneurysms (black arrow), glomerular atrophy (green arrow), sclerosis (orange arrow), and dilated atrophic tubuli containing proteins (blue arrow). Scale bar in (**a**): 100µm.

Figure 5 displays the qualitative grading of glomerulopathy with respect to the proportion of glomeruli affected by the specific pathology. As expected, the histological evaluation showed an increase in the affected glomeruli with age in the ZDF rats with regard to sclerosis, microaneurysms, and completely or partially empty glomeruli [28,29]. In the 12-week-old group, none of the rats had severe glomerulopathy. In the 22-week-old group, one ZDF rat had complete or partial glomerular atrophy in numerous glomeruli, and all of the 22-week-old ZDF rats had microaneurysms in numerous glomeruli. In the 40-week-old group, all of the ZDF rats had affected glomeruli to a severe degree with regard to glomerular atrophy, microaneurysms, and sclerotic areas. The pathological changes of the glomeruli are displayed in Figure 6, showing one field of view of the cortex from the PAS-stained kidney tissue. A more disordered pattern of the cortex was present in the ZDF rats at 40 weeks of age (Figure 6f), with microaneurysms, sclerosis, and either partial or complete glomerular atrophy in numerous glomeruli.

### 3.3. Quantified Measurements from SRUS

**Figure 7:**
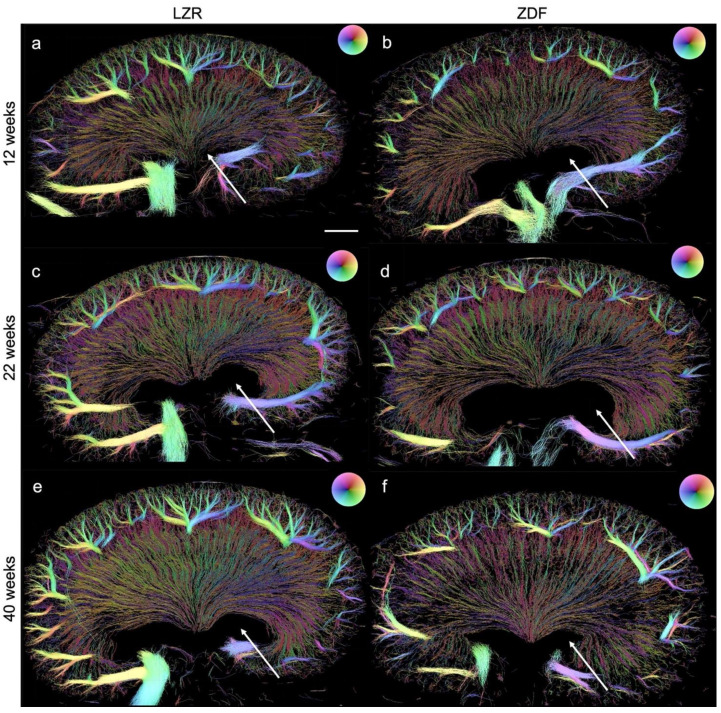
SRUS images of kidneys from the Zucker diabetic fatty (ZDF) rats and the lean Zucker rats (LZRs) by weeks 12, 22, and 40. (**b**,**d**,**f**) illustrate a less dense renal vasculature in the ZDF rats compared with their age-matched LZR (**a**,**c**,**e**). Hydronephrosis (arrows) was found in all the kidneys examined. The direction of the MBs, hence the blood flow’s direction, is illustrated by the color wheel in the upper right corner, e.g., green color represents tracked MBs moving downwards, in the renal vein it correlates with blood flow out of the kidney. Scale bar in (**a**): 2 mm.

**Figure 8:**
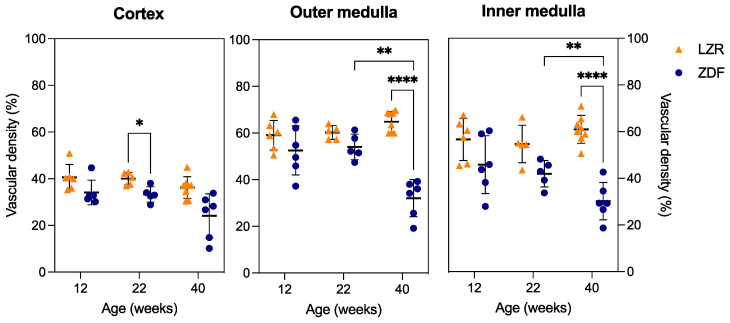
Vascular density measurements from the SRUS images. The vascular density is shown for the cortex, outer medulla, and inner medulla from the lean Zucker rats (LZRs) and the Zucker diabetic fatty rats (ZDF) by weeks 12, 22, and 40. A significant difference is indicated by *p* < 0.05, * 0.01–0.05, ** 0.001–0.01, and **** <0.0001. The lines indicate mean ± SD.

Examples of the SRUS images from each group are presented in Figure 7, showing the presence of hydronephrosis to various extents in all of the kidneys examined. The estimated mean vascular density is illustrated in Figure 8. The ZDF rats had a significantly decreased density in the CO at 22 weeks of age compared to the lean rats’ (*p* = 0.02). The same non-significant trend was seen at week 12 (*p* = 0.19). The high variance in density in the 40-week-old ZDF rats likely influenced the lack of a significant difference (*p* = 0.07). A significant decline in vascular density in the IM and OM of the ZDF rats was present by week 40 (*p* < 0.0001).

**Figure 9:**
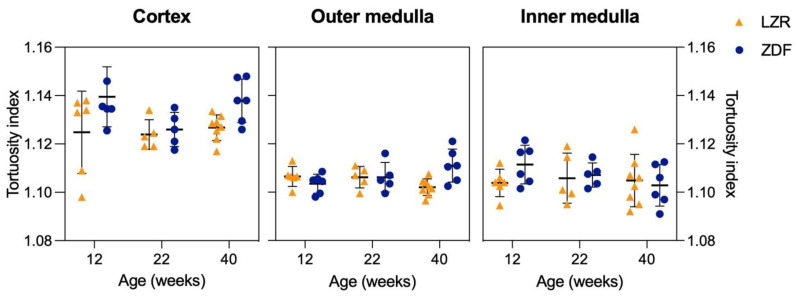
Track tortuosity measurements from the SRUS images. This Figure illustrates the track tortuosity in the cortex, outer medulla, and inner medulla measured in the lean Zucker rats (LZRs) and the Zucker diabetic fatty rats (ZDF) at weeks 12, 22, and 40. The lines indicate mean ± SD.

The mean track tortuosity indices showed no significant differences between any of the groups (Figure 9). However, a tendency towards an increased mean tortuosity in the rats with diabetes was found in all three age groups.

### 3.4. Qualitative and Quantitative Assessment in µCT

**Figure 10:**
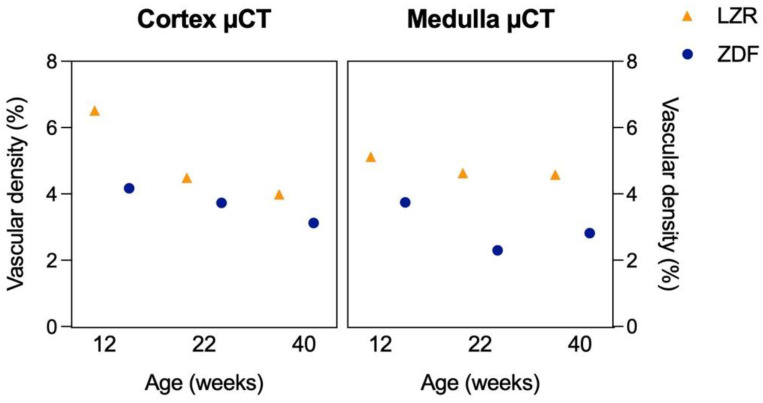
Vascular density measured with the µCT. This Figure shows the vascular density measured in the cortex and medulla of six rats: a lean Zucker rat (LZR) and a Zucker diabetic fatty (ZDF) rat from the 12, 22, and 40 weeks age groups.

The vascular density in the CO and combined IM and OM of three ZDF rats at ages 12, 22, and 40 weeks was consistently lower compared to that of three age-matched LZRs (Figure 10). Additionally, the cortical vascular density appeared to be decreasing with age.

**Figure 11:**
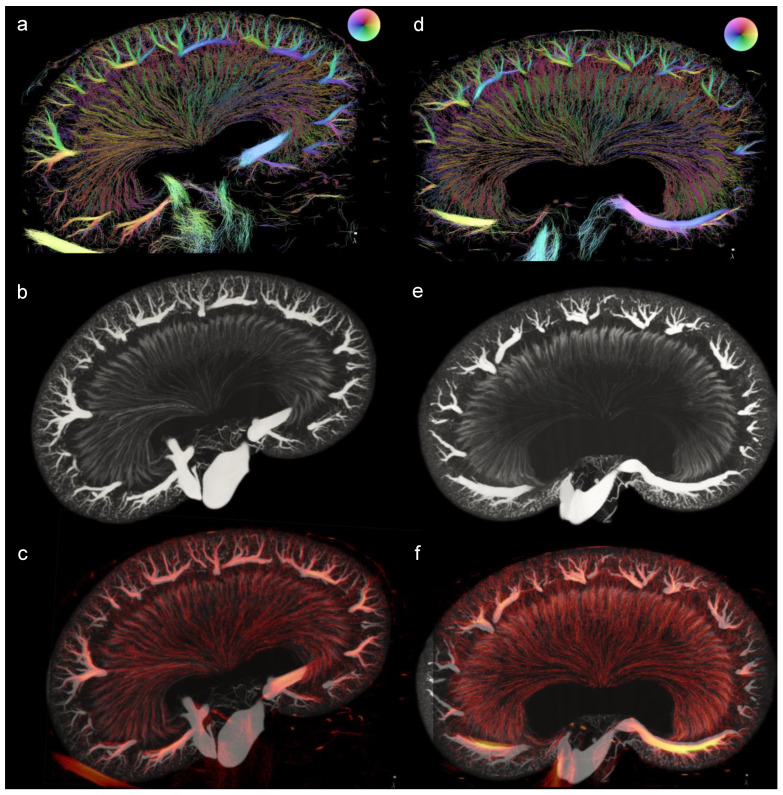
Visual comparison between the µCT and SRUS images. (**a**–**c**) displays the kidney from a lean Zucker rat and (**d**–**f**) the kidney from a Zucker diabetic fatty rat, both at 22 weeks of age. (**a**,**d**) shows the SRUS images, (**b**,**e**) the µCT images (maximum intensity projection of 33 slices), and (**c**,**f**) displays the overlay of the two different modalities. The image slices from the µCT volume were found using Tomviz (version 1.9.0). Due to the transformation of the structure from in vivo to ex vivo, the SRUS images were scaled to match the µCT image in the following way: 50% transparency was used on the intensity of the SRUS image, image (**c**)’s image was rotated 3.45 degrees to the right, increased 5.7% in height, and decreased 3.6% in length. (**f**) was increased 2% in height and decreased 11.5% in length.

A good correspondence between the SRUS and the µCT was found in one kidney from a ZDF rat and one kidney from an LZR, after a coarse manual alignment with a maximum of 11.5% scaling (Figure 11). For example, both imaging modalities clearly displayed the cortical radial arteries as well as the vasa recta in the medulla.

## 4. Discussion

This pre-clinical study investigated alterations in renal blood vessels’ density and vessel tortuosity in T2D ZDF rats as potential predictors of DKD using SRUS. The results confirmed an early onset of diabetes in the ZDF rats, indicated by hyperglycemia at week 12, and an onset of DKD at week 22, indicated by significantly increased ACR levels. Lastly, the histopathological examination showed excessive glomerulopathy in all the ZDF rats by week 40, confirming late-stage DKD. At present, no rodent model exhibiting all three features of human DKD is available [46,47]. Nonetheless, we detected a significant decrease in GFR in the ZDF rats from week 12 to week 40, and both the ACR and the histological examination in particular support the use of this rat model as a late-stage DKD model. The structural vascular parameters from the in vivo SRUS imaging revealed a significant decrease in the cortical vascular density of the ZDF rats at 22 weeks of age. Ex vivo methods have found similar alterations: Maric-Bilkan et al. detected a decrease in the renal vascular density at 12 weeks of age using ex vivo µCT in a type 1 diabetes rat model given streptozotocin, and another study observed a reduced renal vessel area fraction in ob/ob mice (T2D model) at 21 weeks of age measured in histology specimens immunostained with Lectin I [48,49].

The quantitative assessment of vascular density in the µCT images was performed to support the changes in the SRUS-based measurements of vascular density. It was an attempt to automatically quantify vascular density, as manual labeling of vessels is time-consuming and unsuited for larger-scaled studies [15]. An essential feature of µCT imaging is the ability to visualize the whole kidney in 3D. With 2D SRUS imaging, only a single slice of the kidney is visualized. However, the vascular density decrease found through the SRUS imaging corresponded with the µCT-measured vascular density, despite modality differences. Therefore, it seems reasonable to use 2D SRUS density estimations as a proxy measurement for vascular density in the entire kidney. The vascular density was estimated to be higher with the SRUS measurements (~40%) compared to the µCT measurements (~4%) in the CO. This is explained by the intensity threshold used in the µCT measurements, the contrast in the scan, and the image resolution. Additionally, as the µCT is 3D, there is more zero-pixels (pixels without vessels), and, therefore, much lower density indices.

The estimated tortuosity in the SRUS images showed a non-significant trend to increase in the CO and OM of the 40-week-old ZDF rats. Another study found a significantly increased tortuosity in the cerebral microvasculature of aging mice when using the sum of angles metric, which is a different metric than the one used in our study (distance metric) [50]. Shelton et al. quantified the tortuosity on evolving tumors in mice using both metrics and presented an increase in the mean distance metric of ~14% and an increase in the mean sum of angles metric of ~60% compared to the control group [51]. Hence, the chosen metric used seems to be defining for the results obtained.

The renal medulla was affected by hydronephrosis in all the rats except three of the LZRs. Hydronephrosis is defined by trapped urine inside the pelvis, expanding the kidney from the inside out, and it could possibly alter the structure of the medullary vasculature. In humans, hydronephrosis is typically due to obstruction by urolithiasis or infection and is not seen as a direct consequence of diabetes [37]. Hydronephrosis has been described in Zucker rats previously, both in the control rats as well as in the rats with diabetes [41,52,53]. This hydronephrosis may result from inbreeding, as the changes occurred bilaterally; additionally, their relatively high urine output enabled us to rule out obstruction as a cause.

This study has some limitations. The renal structural alterations were quantified with SRUS to detect the initiation of DKD through decreased vascular density before an increase in albuminuria. A tendency towards a decline in the cortical vascular density was found at week 12, with presence of an outlier in the ZDF group. A ROUT outlier analysis with a Q-value of 1% was performed on all the groups, which identified one outlier in the 12-week-old ZDF group. Besides the higher cortical vascular density in this ZDF rat, a particular, lower ACR was measured (16,098 mg/g) compared to the average ACR for the rest of the 12-week-old ZDF rats (42,789 mg/g). This speaks for the exclusion of this particular rat. Excluding the outlier resulted in a significant difference in cortical vascular density (*p* = 0.04) at week 12. This suggests that a higher sample size could have allowed us to show a significant decrease in the vascular density earlier. Furthermore, the afferent/efferent arterioles, the glomeruli, and the peritubular capillary network of the cortical microvasculature are not visible with our current MB tracking algorithm (Zoomed image, Figure 11). The lack of microvascular visualization in the renal cortex could also have influenced the detection of earlier vascular alterations, as vascular rarefaction usually affects the capillaries and arterioles first [54]. This also speaks to MB tracking being a challenge in the renal cortex, as the vessels are very tortuous and densely organized.

In a recent systematic review from 2020 on microvasculature investigated in humans and animals with chronic kidney disease (CKD), it was concluded that most microvascular rarefaction research has focused on the heart [55]. Moreover, the review in question showed that most vascular studies have been devoted to macrovascular complications. One reason for this focus could be that individuals with CKD have a higher risk of mortality from cardiovascular disease than a risk of developing kidney failure [56,57]. However, macrovascular disease has been suggested to be ‘the tip of the iceberg’ when correlated to what is clinically measurable, and the affection of the microcirculation is considered a silent, subclinical process [55]. Focusing on detecting kidney injury prior to the current clinical measurements could potentially increase survival due to earlier diagnosis and initiation of treatment. In the clinical settings at present, no imaging modality is available to visualize renal microvasculature in vivo. Contrast-enhanced ultrasound (CEUS), used in clinics, is often unable to distinguish early kidney disease from the healthy kidney; instead, the MB pattern is used to differentiate solid renal tumors from pseudo-tumors [58]. Zhang et al. showed no significant differences in renal perfusion using CEUS on a streptozotocin-induced DKD rat model compared with healthy controls [23]. Other imaging modalities have shown to be limited in resolution as well. Von Stillfried et al. quantified the renal microvascular rarefaction using CD31 antibody staining, showing a significant cortical vascular density reduction by 59% in individuals with CKD [59]. This was further investigated using post mortem CT angiography, showing superiority to in vivo CT angiography; however, only the vessels down to the level of arcuate arteries was visualized. These ex vivo quantifications underline the need for a high-resolution imaging tool in order to be able to assess early DKD in vivo to prevent disease progression.

To make SRUS imaging clinically beneficial, future studies must progress to enable transcutaneous scanning. Currently, transcutaneous SRUS scans are challenging due to out-of-plane motion, as demonstrated in a human breast cancer study by Dencks et al. [60]. The out-of-plane motion would be solved with SRUS imaging in 3D, as shown in another study of a rat brain using a matrix probe [61]. The transcutaneous application would enable sequential measurements on SRUS data from the same rat to accommodate the present time-gap in measurements from week 12 to week 22 in this current study. Furthermore, it is crucial to investigate the possibilities of generating images at an even higher resolution for visualization of the entire cortical renal microvasculature. This could possibly be achieved using an ultrasound probe with a higher frequency.

## 5. Conclusions

This study showed that it was possible to detect a decrease in cortical vascular density in early DKD development using in vivo imaging. This is the first step towards SRUS becoming a tool for monitoring and prognosing DKD in the future.

## 6. Patents

The algorithm for tissue motion correction used in this study is patented by J.A.J. and I.T.

## Figures and Tables

**Table 1:** Basic characteristics and biochemical biomarkers for kidney injury.

	12 Weeks		22 Weeks		40 Weeks	
Basic characteristics	LZR (*n* = 6)	ZDF (*n* = 6)	LZR (*n* = 5)	ZDF (*n* = 5)	LZR (*n* = 8)	ZDF (*n* = 6)
Body weight (g)	276 ± 12.74	361 ± 19.26 *	371 ± 15.22	405 ± 14.15 *	424 ± 21.09	405 ± 69.80
Kidney weight (g)	1.28 ± 0.09	1.67 ± 0.09 *	1.35 ± 0.08	2.20 ± 0.46 *	1.57 ± 0.20	2.41 ± 0.29 *
KW/BW (%)	0.46 ± 0.04	0.46 ± 0.02	0.37 ± 0.02	0.54 ± 0.10 *	0.35 ± 0.04	0.60 ± 0.07 *
Mean arterial pressure (mmHg)	104 ± 13.26	118 ± 15.52	110 ± 9.67	119 ± 14.69	110 ± 9.68	100 ± 16.65
Biochemical biomarkers						
Blood glucose (mmol/L)	6.17 ± 0.37	22.97 ± 5.28 *	6.48± 0.55	30.8 ± 0.78 *	7.2 ± 0.53	28.23 ± 8.84 *
P-creatinine (mg/dL)	2.08 ± 0.59	1.33 ± 0.17 *	2.03 ± 0.26	1.54 ± 0.51	1.88 ± 0.30	2.76 ± 2.09
Urinary creatinine (mg/dL)	72.92 ± 33.09	14.50 ± 6.21 *	75.60 ± 36.84	9.89 ± 2.69 *	28.66 ± 15.67	5.09 ± 2.53 *
Urinary albumin (mg/mL)	0.66 ± 0.67	2.41 ± 1.48 *	1.59 ± 1.21	1.29 ± 0.40	0.62 ± 0.66	4.78 ± 3.27 *
Creatinine excretion rate (µg/min)	4.90 ± 1.51	2.55 ± 0.40	2.14 ± 0.61	3.12 ± 0.78	2.76 ± 3.36	4.56 ± 6.73
Albumin excretion rate (µg/min)	5.21 ± 2.28	10.29 ± 8.67	4.89 ± 3.44	41.72 ± 14.48 *	4.85 ± 5.11	148.93 ± 101.67 *
Albumin/creatinine ratio (mg/g)	32,114 ± 15,416	38,341 ± 27,739	21,729 ± 14,143	138,633 ± 52,788 *	23,497 ± 27,843	1,014,106 ± 468,583 *
GFR/Creatinine clearance (mL/min)	0.11 ± 0.10	0.19 ± 0.03	0.10 ± 0.02	0.23 ± 0.11	0.14 ± 0.15	0.07 ± 0.06
Diuresis (µL/min)	3.52 ± 3.71	20.47 ± 8.92 *	3.28 ± 1.71	32.75 ± 9.65 *	8.84 ± 7.25	36.63 ± 26.72 *

Presenting the Zucker diabetic fatty (ZDF) rats and the lean Zucker rats (LZRs) at 12, 22, and 40 weeks of age. The right kidney was cut in half before weighing to prevent the urine in the hydronephrotic cavity from affecting the measurement. Values are means ± SD. * denotes *p* < 0.05 versus the age-matched LZR.

## Data Availability

The analysis algorithms and processed data for SRUS can be accessed upon request.
